# Direct analysis of volatile organic compounds in foods by headspace extraction atmospheric pressure chemical ionisation mass spectrometry

**DOI:** 10.1002/rcm.7975

**Published:** 2017-10-11

**Authors:** P. Perez‐Hurtado, E. Palmer, T. Owen, C. Aldcroft, M.H. Allen, J. Jones, C.S. Creaser, M.R. Lindley, M.A. Turner, J.C. Reynolds

**Affiliations:** ^1^ Centre for Analytical Science, Department of Chemistry Loughborough University Ashby Road Loughborough LE11 3TU UK; ^2^ Advion Ltd Kao Hockham Building, Edinburgh Way Harlow CM20 2NQ UK; ^3^ Advion Inc. 10 Brown Road, Suite 101 Ithaca NY 14850 USA; ^4^ School of Sports, Exercise and Health Sciences Loughborough University Ashby Road Loughborough LE11 3TU UK

## Abstract

**Rationale:**

The rapid screening of volatile organic compounds (VOCs) by direct analysis has potential applications in the areas of food and flavour science. Currently, the technique of choice for VOC analysis is gas chromatography/mass spectrometry (GC/MS). However, the long chromatographic run times and elaborate sample preparation associated with this technique have led a movement towards direct analysis techniques, such as selected ion flow tube mass spectrometry (SIFT‐MS), proton transfer reaction mass spectrometry (PTR‐MS) and electronic noses. The work presented here describes the design and construction of a Venturi jet‐pump‐based modification for a compact mass spectrometer which enables the direct introduction of volatiles for qualitative and quantitative analysis.

**Methods:**

Volatile organic compounds were extracted from the headspace of heated vials into the atmospheric pressure chemical ionization source of a quadrupole mass spectrometer using a Venturi pump. Samples were analysed directly with no prior sample preparation. Principal component analysis (PCA) was used to differentiate between different classes of samples.

**Results:**

The interface is shown to be able to routinely detect problem analytes such as fatty acids and biogenic amines without the requirement of a derivatisation step, and is shown to be able to discriminate between four different varieties of cheese with good intra and inter‐day reproducibility using an unsupervised PCA model. Quantitative analysis is demonstrated using indole standards with limits of detection and quantification of 0.395 μg/mL and 1.316 μg/mL, respectively.

**Conclusions:**

The described methodology can routinely detect highly reactive analytes such as volatile fatty acids and diamines without the need for a derivatisation step or lengthy chromatographic separations. The capability of the system was demonstrated by discriminating between different varieties of cheese and monitoring the spoilage of meats.

## 1 INTRODUCTION

The development of new interfaces that enable direct analysis of volatile organic compounds (VOCs) represents an attractive and low‐cost alternative for rapid analysis. Currently, there are a significant number of analytical techniques for VOCs, which include methods such as chemical sensing (electronic noses),^1^, ^2^ ion mobility spectrometry,^3^, ^4^ and optical spectroscopy.^5^ However, mass spectrometry remains the gold standard technique enabling chemical specificity of detection.

Mass spectrometry techniques used for the analysis of VOCs include gas chromatography/mass spectrometry (GC/MS),^6^, ^7^, ^8^, ^9^ selected ion flow tube mass spectrometry (SIFT‐MS),^10^, ^11^, ^12^, ^13^ proton transfer reaction mass spectrometry (PTR‐MS),^14^, ^15^ and ion‐molecule reaction mass spectrometry (IMR‐MS).^16^ GC/MS provides excellent results offering high chromatographic resolution and straightforward coupling with pre‐concentration techniques such as thermal desorption.^17^ However, GC/MS has a number of drawbacks including the need for molecular derivatisation of reactive functional groups such as carboxylic acids and amines and potentially long chromatographic runs (30–60 min) for complex analyte mixtures. Real‐time mass spectrometry techniques such as PTR‐MS and SIFT‐MS have also been used for this type of analysis, and their potential for monitoring volatiles in real‐time has been proven.^18^, ^19^, ^20^, ^21^ In addition, the development of innovative methods using standard ambient ionisation sources such as atmospheric pressure chemical ionisation (APCI) and electrospray ionisation (ESI) has considerably increased in recent years.^22^, ^23^, ^24^, ^25^, ^26^, ^27^, ^28^, ^29^ These ionisation sources allow for the interfacing of volatile analysis with mass spectrometry without requiring specialised instrumentation limited to analysing VOCs. For example, APCI has been widely used for the analysis of volatiles.^26^, ^27^, ^29^, ^30^, ^31^ Extractive electrospray ionisation (EESI) has also been used to analyse volatiles in different matrices including beer,^32^ breath,^23^, ^24^, ^29^, ^33^ an active pharmaceutical ingredient,^34^ and fragrances.^35^ This ionisation method has also enabled the detection of non‐volatile compounds such as nicotine,^25^ urea,^24^ and creatinine.^36^

The work presented here describes the design and construction of an interface for the direct introduction of VOCs into the APCI source of a compact quadrupole mass spectrometer. This modification accommodates the introduction of volatiles using a new jet‐pump designed in‐house. The pump operates on the basis of the Venturi effect, which enables the introduction of volatiles into the mass spectrometer *via* the APCI gas line. The suitability of the interface for a variety of qualitative and quantitative applications is demonstrated. Volatile profiles for compounds such as fatty acids and biogenic amines, which are challenging to analyse by GC/MS without derivatisation, were obtained with minimal effort using our design.

## 2 APPLICATIONS

### 2.1 Food

The food industry has shown a particular interest in rapid analysis to differentiate the freshness of different foods by measuring the release of specific volatiles.^4^, ^13^, ^37^ Currently, there is also a high demand for new methods to screen meat products from supermarket chains, which would enable discrimination between different types of meat and the identification of adulteration. Ambient ionisation methods such as liquid extraction surface analysis/mass spectrometry (LESA/MS)^38^ and rapid evaporative ionisation mass spectrometry^39^ have been implemented for this purpose, enabling rapid *in situ* detection, and have proven to be useful in authenticating meat products. In this application different cheese samples and meat samples were used to demonstrate the capabilities of the interface for fatty acid and biogenic amine profiling in the gas phase.

### 2.2 Forensics

The characterisation of volatile profiles that identify human remains is of significant interest in forensic research. It is widely accepted that body decomposition starts shortly after death.^40^ Part of the decomposition process is constituted by the self‐digestion of cells, where proteins are released and broken down by bacteria into their building blocks. From these processes several compounds are released, amongst these are putrescine, cadaverine, phenol, indole, butanol, and octanal.^41^ Pig carcasses have been proposed as human body analogues to serve as training aids for human remains detection dogs.^41^ Therefore, pork has been selected for the decomposition studies presented here. This particular application focused on monitoring the production of indole in decomposing meat due to deamination of tryptophan.

## 3 EXPERIMENTAL

### 3.1 Materials

Volatile fatty acids, indole, cadaverine, putrescine, formic acid, ammonium acetate, methanol, acetone, ethanol, and water were acquired from Sigma Aldrich Ltd (Gillingham, UK). Food samples were acquired from a local supermarket. Standards were used to tune the instrument, optimise the conditions for each experiment and confirm the identity of analytes.

### 3.2 Compact mass spectrometer: direct VOC sampling interface

**SCHEME 1:**
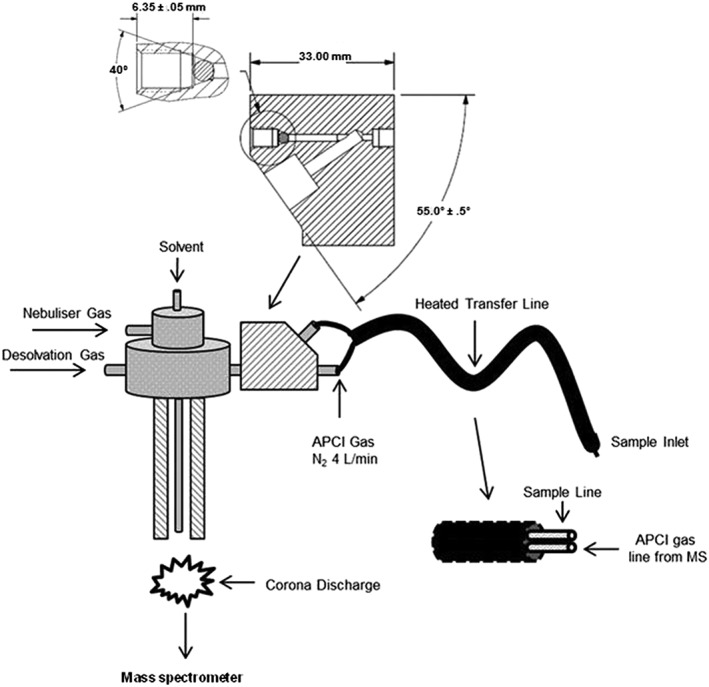
Schematic diagram of the compact mass spectrometer APCI source. The APCI gas line and sample inlet line were accommodated inside the heated transfer line and directed to the Venturi pump

Experiments were performed using an in‐house designed Venturi pump coupled to the APCI probe of an Expression single quadrupole compact mass spectrometer (Scheme 1) from Advion, Inc. (Ithaca, NY, USA). The gas line, sample inlet and jet pump were heated to 180°C using a commercially available heated transfer line (Quantitech Ltd, Milton Keynes, UK). The APCI nitrogen gas supply flow rate was adjusted to 4 L/min, which gave a measured suction from the sample inlet of over 500 mL/min. The instrument was operated in both negative and positive ionisation mode depending on the target analytes. Table 1 shows the mass spectrometer conditions used for the studies reported here. All the spectra were acquired within the range of *m/z* 30–300 and blank measurements were acquired for each case.

**Table 1:** Compact mass spectrometer direct sampling interface conditions for each study

Parameter	Cheese	Pork
APCI gas (N_2_) flow rate (L/min)	4	4
Measured suction from Venturi pump (mL/min)	>500	>500
Capillary temperature (°C)	250	250
Source voltage (V)	15	15
APCI gas (N_2_) temperature (°C)	350	350
Corona discharge voltage (kV)	5	5
Capillary voltage (V)	150	60
Ionisation mode	Negative	Positive

### 3.3 Optimization of the interface

The principal criteria considered in the development of the volatile interface were that the condensation of volatiles onto cold surfaces should be prevented and that the interface can be readily coupled to the APCI source of the compact mass spectrometer. To address the first criterion the system was heated above 70°C using nitrogen gas heated by a commercially available heating jacket (Quantitech), see Scheme 1. In order to connect the Venturi pump to the APCI source a bespoke connector was used. The collection of volatiles was achieved by using a 1/8" silico‐steel sampling line of approximately 1 m length which was attached directly to the Venturi pump. The flexibility of the line allows for easy sampling. A mixture of acetone and calibration components (Agilent APCI/APPI tuning mixture, PN: G2432A; Agilent Technologies, Stockport, UK) was used to calibrate the system.

### 3.4 Temperature studies

Temperature proved to be a critical variable in the development of the interface. The optimisation was carried out by setting the sample line at temperatures of 40°C, 70°C and 100°C. The latter temperature was shown to be the optimal temperature across a number of applications, since it prevented moisture from building up in the transfer line.

### 3.5 Sampling

#### 3.5.1 Cheese study

Four different cheeses, Red Leicester, Wensleydale, Blue Stilton and Goats, were chosen for this study. The samples were cut into 1 × 1 cm sections. Approximately 2 g of each cheese was placed into a 20‐mL headspace vial. The samples were heated to an optimal temperature of 70°C for 2 h. Sampling was achieved by connecting the headspace vial to the sample inlet as shown in Scheme 1. To promote formation of negative ions, 50:50 methanol/water +10 mM ammonium acetate was infused into the APCI source at a rate of 10 μL/min. Samples were analysed for 5 min; a blank vial was analysed for 5 min prior to each analysis to enable subtraction of the background from the sample mass spectra.

#### 3.5.2 Pork decomposition study

Samples of pork were mixed to evenly distribute any bacteria. The meat was cut into approximately 2 × 2 cm sections. Three replicates of approximately 2 g each were introduced into 20‐mL silanized headspace vials (Phenomenex Ltd, Macclesfield, UK). The vials were left constantly open to atmospheric conditions, and crimped 2 h prior to sampling the headspace. The experiment was performed over the course of 9 days at room temperature (~27°C) with no previous sample treatment.

### 3.6 Multivariate analysis

Spectra were obtained using the experimental conditions described above. Mass spectra were extracted from the Advion Data Express software as .csv files and then processed using Microsoft Excel 2010, and a sum of the intensities of each mass was calculated. The first 5 min of data collection were then subtracted from the last 5 min to remove the background. For multivariate statistical analysis, six separate samples of each variety were analysed. Samples were analysed over a 4‐day period with one variety of cheese being analysed on each day. In addition, to test the inter‐day reproducibility of the system, a sample of Red Leicester cheese was analysed after each set of samples as a pseudo‐quality control sample.

### 3.7 Quantitative performance of the interface

**SCHEME 2:**
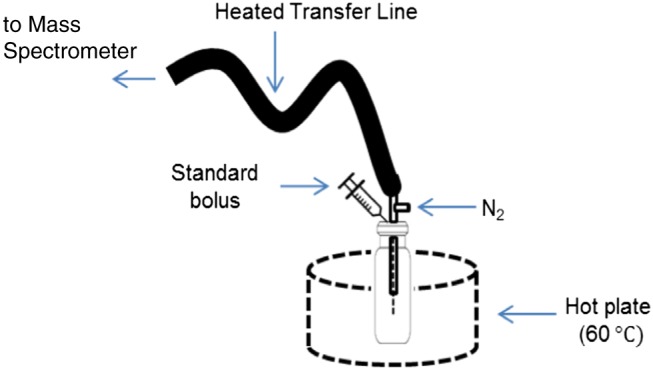
Nitrogen‐regulated hot vials. A shot of 1 mL of standard was introduced into the vials set at 60°C [Color figure can be viewed at wileyonlinelibrary.com]

The quantitative performance of the interface was tested using an indole standard. A calibration curve was generated using liquid solutions of indole in methanol at concentrations ranging from 1 to 60 ppm. Sampling was achieved by spiking 1 mL of the calibration solutions into nitrogen‐regulated hot vials (Scheme 2). Three measurements were taken for each solution.

## 4 RESULTS AND DISCUSSION

**Figure 1:**
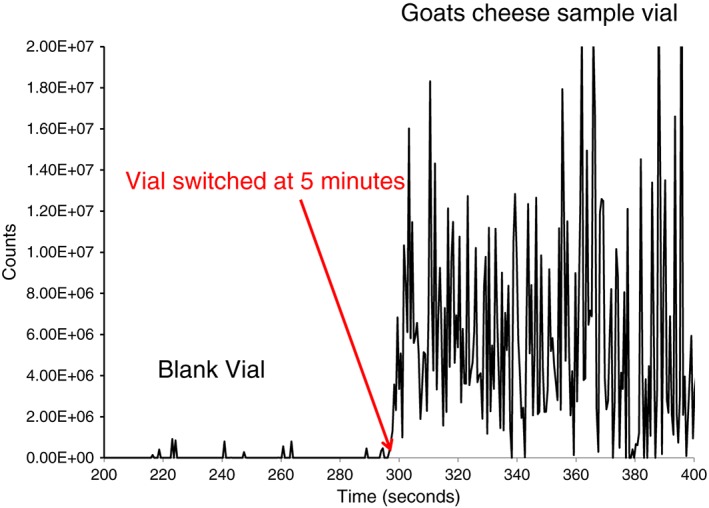
Extracted ion chromatogram of the [M − H]^−^ ion of octanoic acid (*m/z* 143), when changing from blank vial to sample vial at 300 s [Color figure can be viewed at wileyonlinelibrary.com]

The Venturi pump–APCI‐MS interface allowed the rapid, direct analysis of VOCs for all the samples. Using the conditions described above, the sample residence time in the heated transfer line and Venturi pump was less than 1 s, giving an almost instantaneous response on the mass spectrometer when a vial containing a sample was analysed. This potentially allowed very short run times and high sample throughput. The rapid response rate of the instrument is highlighted in Figure 1, which follows the extracted ion trace of the [M − H]^−^ ion of octanoic acid (*m/z* 143) from a Goats Cheese sample. As we switched from a blank vial to the sample vial at 300 s the increase in the response from the *m/z* 143 ion occurred immediately and did not show a gradual increase due to interactions with the walls of the sampling apparatus. A high degree of signal variability was observed in the ion chromatogram for *m/z* 143. This variation was observed for all of the VOCs measured directly from foods in this study and probably occurred because of variable rates of VOC release from the un‐homogenised food samples. In addition using these conditions minimal sample carryover was observed between samples when the sample line assembly was maintained at 100°C even with the some of the larger and less volatile species (such as octadecanoic acid) analysed in these experiments. The system has been tested with difficult compounds and two different applications of this versatile design will be discussed in the following sections.

### 4.1 Detection of fatty acids in cheese

**Figure 2:**
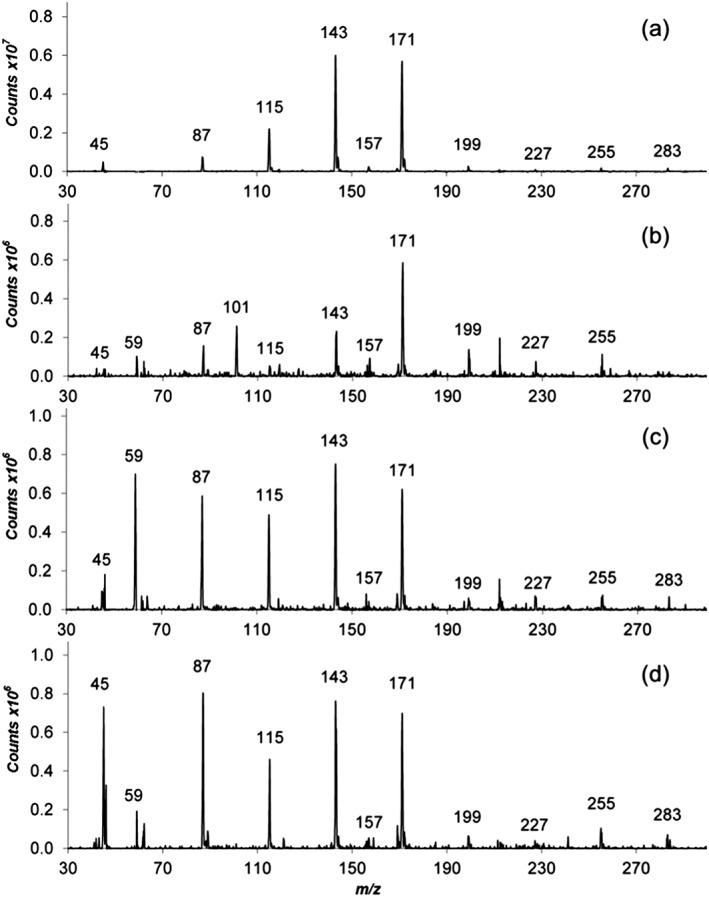
Full scan mass spectra of the volatile profile from (A) Goats Cheese, (B) Blue Stilton, (C) Red Leicester, and (D) Wensleydale cheese

Real‐time screening of volatiles in cheese was possible with no sample pre‐treatment. The cheese headspace was directly analysed from the vials using the volatiles interface. Figure 2 shows the full scan mass spectrum of the volatile profile obtained for the selected cheeses. Common volatiles found in all the samples are shown in Table 2. The most significant difference between samples was the presence of pentanoic acid ([M − H]^−^ ion, *m/z* 101) in Blue Stilton, which gives the characteristic smell of this particular cheese. In addition, no [M − H]^−^ ion for acetic acid (*m/z* 59) was detected in Goats Cheese. The [M − H]^−^ ions of common acids such as butanoic (*m/z* 87), hexanoic (*m/z* 115), octanoic (*m/z* 143), nonanoic (*m/z* 157), decanoic (*m/z* 171) and hexadecanoic (*m/z* 255) were found across the selection of cheese samples. This acid content in cheese has been previously reported in the literature.^42^ Other approaches have been reported for the direct analysis of the headspace of cheese samples.^43^ However, the system reported here has the advantages of the direct identification of organic acids using APCI, which produces [M − H]^−^ ions for each compound and has no requirement for a derivatisation step as in GC/MS analysis. Unsaturated fatty acid species with chain lengths of up to 18 carbons (stearic acid) were observed in the APCI‐MS spectra of all the cheeses analysed in this study. Stearic acid has a melting point of 69.3°C and a boiling point of 361°C, thus demonstrating the capability of the interface for analysing semi‐volatile compounds.

**Table 2:** Mass list of analytes found across the different cheeses

Fatty acid	Formula	MW	[M − H]^−^	Measured *m/z*
Formic acid	CH_2_O_2_	46.0	45.0	45.0
Acetic acid	C_2_H_4_O_2_	60.0	59.0	59.0
Butanoic acid	C_4_H_8_O_2_	88.1	87.1	87.1
Hexanoic acid	C_6_H_12_O_2_	116.1	115.1	115.2
Octanoic acid	C_8_H_16_O_2_	144.2	143.2	143.2
Nonanoic acid	C_9_H_18_O_2_	158.2	157.2	157.2
Decanoic acid	C_10_H_20_O_2_	172.3	171.3	171.2
Tetradecanoic acid	C_14_H_28_O_2_	228.4	227.4	227.3
Hexadecanoic acid	C_16_H_32_O_2_	256.4	255.4	255.3
Octadecanoic acid	C_18_H_36_O_2_	284.5	283.5	283.3

### 4.2 Multivariate statistical analysis

Following APCI‐MS analysis the cheese varieties were compared against each other individually using a supervised partial least‐squares discriminant analysis approach. This approach forces a separation between the two groups and enables the construction of s‐plots which can be used to identify the variables which contribute most to the separation. This enabled the construction of a 339 variable model (Table S1, supporting information) that reflected the differences between each of the four cheese varieties.

**Figure 3:**
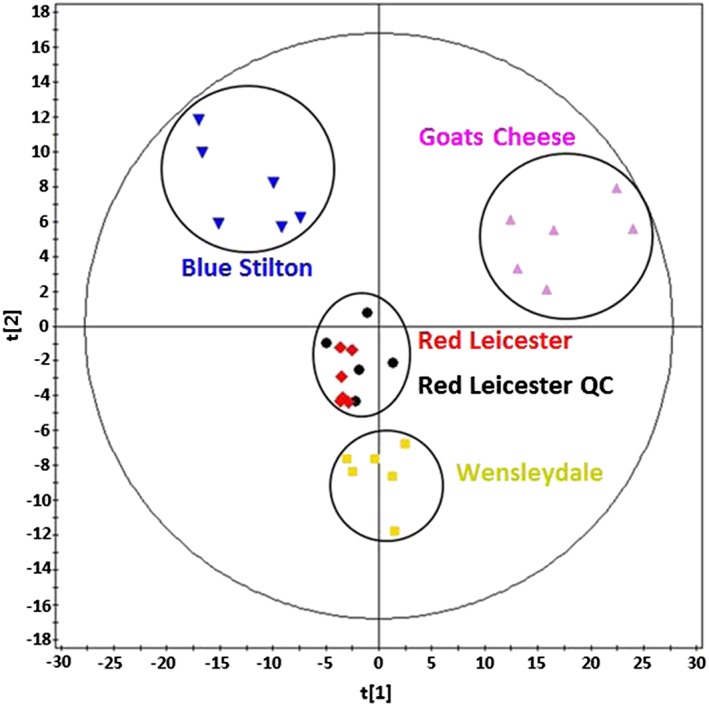
Principal component analysis of the four cheese varieties [Color figure can be viewed at wileyonlinelibrary.com]

**Figure 4:**
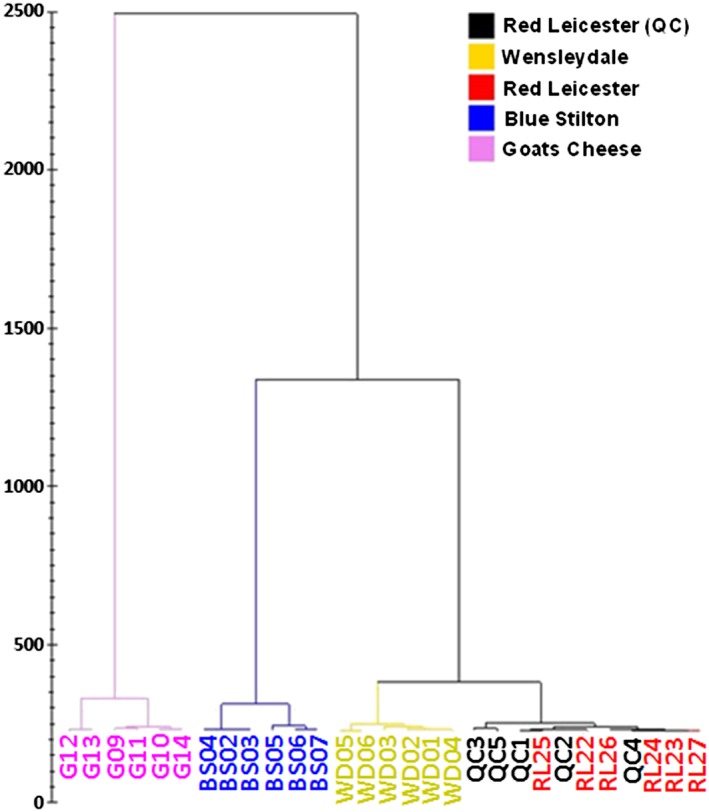
Hierarchical cluster analysis of the four cheese varieties with Red Leicester QC samples included [Color figure can be viewed at wileyonlinelibrary.com]

This model was then used to perform an unsupervised principal component analysis (PCA) separation of all samples from four of the different cheese varieties (and the pseudo‐QC samples). The separation obtained from this is shown in Figure 3. This shows a clear separation of all four cheese varieties with no overlap, demonstrating that the system is capable of analysing complex food samples and differentiating between different varieties. The grouping of the Red Leicester QC samples which were run over 5 subsequent days after sampling other cheese varieties shows closing grouping with the other Red Leicester samples, indicating that the reproducibility of the approach and the statistical model is sound. The hierarchical cluster analysis plot obtained (Figure 4) confirms the separation observed in the PCA plot with each of the cheese varieties in their own individual cluster. It can be noted from Figure 3 that the highest degree of separation is observed between the Goats Cheese and the other three cheeses which are made using cow's milk. The Blue Stilton is then the next best resolved from the less mature cheeses. The Wensleydale and Red Leicester cheeses cluster closest together which is expected as they are the most similar.

### 4.3 Detection of biogenic amines in pork

**Figure 5:**
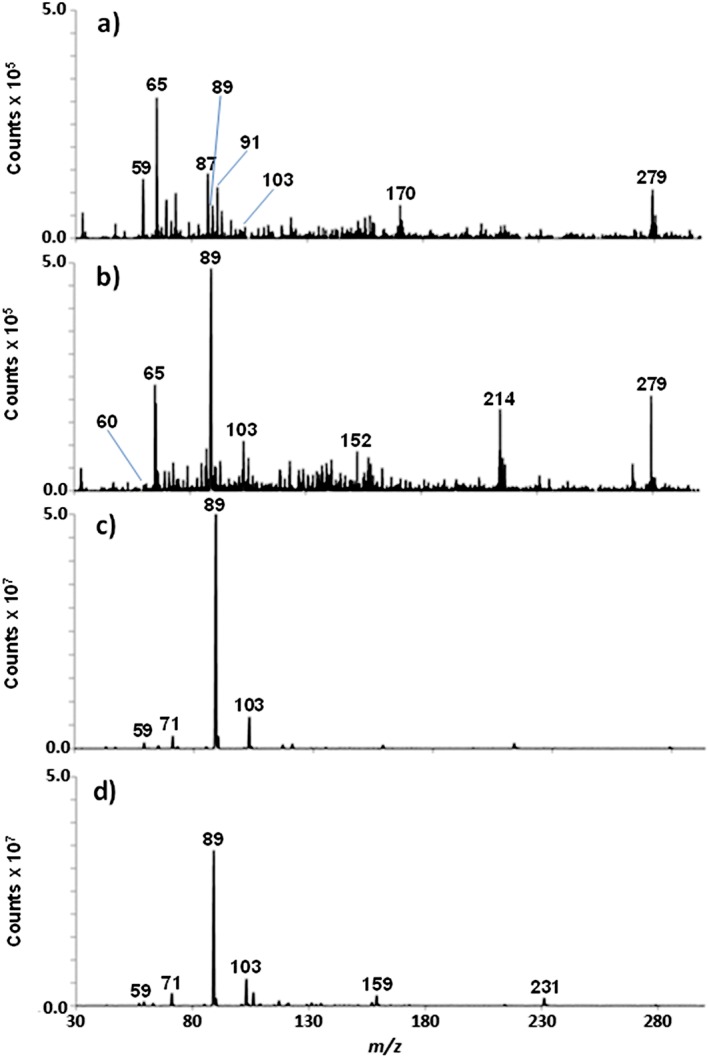
Full scan APCI mass spectra of the volatile profiles of a decomposing pork sample from (A) day 0, (B) day 1, (C) day 2, and (D) day 3 [Color figure can be viewed at wileyonlinelibrary.com]

**Figure 6:**
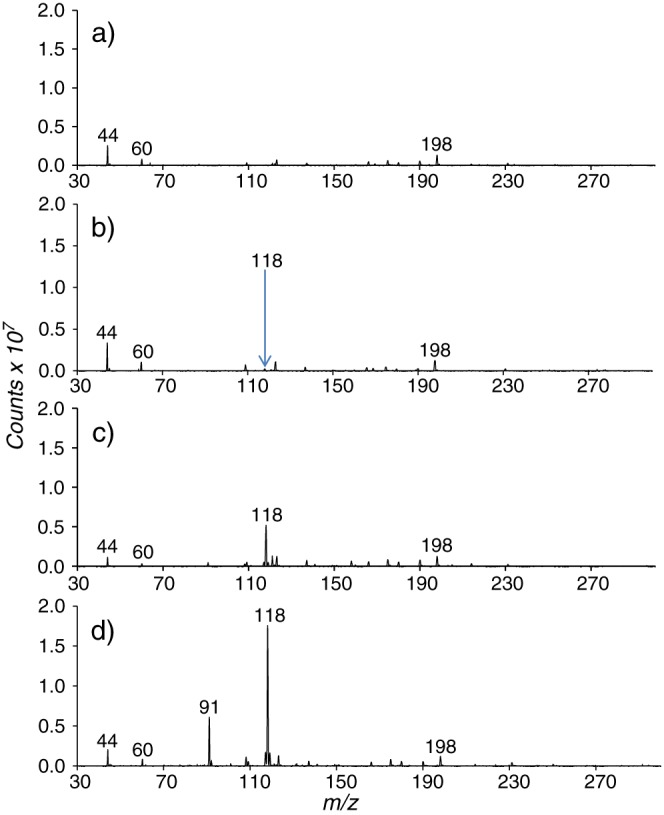
Full scan mass spectra of decomposing pork on (A) day 4, (B) day 5, (C) day 6, and (D) day 7 [Color figure can be viewed at wileyonlinelibrary.com]

Another group of compounds that are challenging to analyse using gas chromatography are biogenic amines. Diamines such as putrescine (1,4‐diaminobutane) and cadaverine (1,4‐diaminopentane) have low vapour pressures at room temperature. However, at physiological pH they are commonly present in the sample as involatile salts and not present in the more volatile free‐base structure. They are also very reactive, which leads to peak symmetry issues and significant losses in the injection liner in GC/MS analysis. Due to this behaviour, a derivatisation step would be mandatory if GC/MS was to be used to analyse these species. However, using the direct VOC analysis interface for the compact mass spectrometer it was possible to detect putrescine and cadaverine with minimal effort and Figure 5 shows that these species can be clearly observed in the positive ion mode at *m/z* 89 and 103, respectively, as [M + H]^+^ ions. It is important to mention that in order to detect these diamines a silico‐steel sample line was used which had been pre‐conditioned at 40°C in a vacuum oven. During the course of the first 4 days of decomposition three biogenic amine species were detected. Putrescine and cadaverine were detected at day 0 and observed to increase dramatically over the next 2 days. The highest response for these diamines was observed at day 2 and it began to decline after. Trimethylamine was also detected at *m/z* 60 (Figures 4 and 5). This amine is highly volatile compared with the other amines, which thus have lower vapour pressures at room temperature, and it is observed at low levels in all the spectra obtained after day 1. In some pork samples aged for a longer period (Figure 6) a peak assigned to indole was also reliably detected after 6 days of decomposition. Figure 5 shows the mass spectrum of decomposed pork over a 4‐day period, from day 4 onwards, showing the evolution of the [M + H]^+^ ion of indole at *m/z* 118. Assignment of the *m/z* 118 ion was confirmed by direct comparison with an indole standard (Figure S1, supporting information); the indole standard also yielded an ion at *m/z* 91 which was observed to increase in parallel with the [M + H]^+^ ion (Figure 6). This ion results from a loss of HCH from the protonated indole, its presence in both the standard and the pork samples providing support for the assignment of *m/z* 118 as the indole [M + H]^+^ ion. The presence of indole in decomposing pork has been previously reported^41^ supporting the findings reported here. This ion, seen to increase significantly after day 4 of the decomposition, could originate either from the action of indole producing bacteria or from direct breakdown of the tissue. Repeat analysis of multiple pork samples (n = 5) showed that indole was observed at high levels in three samples after 5 days of aging but was absent in the other two samples. This suggests that this species is probably produced by bacterial metabolism as the storage conditions were the same for all of the samples, suggesting that indole‐forming bacteria were present in high levels in three of the pork samples but not in the other two samples.

### 4.4 Quantitation of indole in decomposing pork

The volatiles interface developed in this study is completely open to the atmosphere. As a consequence, the humidity in the source can change depending on the laboratory environment, and other species present in the laboratory air can interact with and potentially suppress the formation of specific analyte ions in the APCI source. Therefore, a more regulated environment was needed to control external interferences that might suppress the signal of the analyte. Passing a flow of nitrogen through the vial instead of pulling laboratory air through, using the nitrogen regulated hot vial arrangement shown in Scheme 2, enabled a quantitative response to be obtained for indole standards (Figure S2, supporting information). Without the nitrogen flow present a linear calibration could not be obtained, demonstrating clearly the effect of uncontrolled external interfering factors on the APCI source. Sampling was achieved by spiking 1 mL of the indole standard solutions into nitrogen‐regulated hot vials, and three replicates were measured for each solution. A linear calibration curve was obtained for concentrations between 1 and 60 μg/mL with an R^2^ value of 0.9918. The average signal‐to‐noise ratio obtained from the three repeat measurements at the 1 μg/mL level (Figure S3, supporting information) was 7.6:1 indicating limits of detection and quantification of 0.395 μg/mL and 1.316 μg/mL, respectively. Using the calibration obtained in Figure S2 (supporting information) we can determine that indole is present at 14.1 μg/g pork at day 6 and at 51.8 μg/g pork at day 7. The level of indole present at day 5 is too low to be quantified by the system. Please note that these figures assume that 100% of the indole is extracted from the pork. This is unlikely with a complex matrix and the level of indole in the pork may be higher than these results; however, this does demonstrate the capability of the system to quantitatively monitor the level of indole over the 7‐day period. The linear dynamic range of the system can potentially allow for quantitation of the volatiles. However, care needs to be taken by operating the system under regulated conditions. Another problem that may arise using the external standardisation shown here is that, without any pre‐separation of analytes passing through into the source, ion suppression effects may impact negatively on the quantitative performance. This would particularly affect low proton affinity analytes as other species present with higher proton affinity will sequester the charge in the ion source. Indole has a relatively high proton affinity of 933.4 kJ/mol^44^ and its [M + H]^+^ ion is observed to dominate the spectrum in Figures 6c and 6d, so this is not expected to have a significant impact in this example However, these issues can be addressed for targeted analytes through the use of an isotopically labelled standard, while maintaining the fast analysis times of the technique.

## 5 CONCLUSIONS

The direct analysis interface system is a simple and cost‐effective alternative to traditional techniques for the introduction of volatiles into the mass spectrometer. The capability of the system for direct analysis of fatty acids and biogenic amines from decomposing meat samples was successfully demonstrated. These analytes, which are highly reactive and problematic to analyse directly with traditional methodologies such as GC/MS, were detected without the need for any derivatization reaction or chromatographic separation. The system is shown to be capable of discriminating between different varieties of cheese using an unsupervised principal component analysis model highlighting a potential application of the deployable instrumentation in food authenticity and provenance testing. Quantitative analysis was also achievable using external standardisation for indole and is shown to be capable of limits of detection in the ng/mL range, but only when the system was under controlled headspace conditions. Due to the absence of a separation method, competitive ionisation effects in the ion source will also limit the quantitative performance of this approach; however; this issue can be overcome in targeted studies by using an isotopically labelled internal standard.

The example applications demonstrated here display the versatility of this approach for the rapid and semi‐quantitative analysis of VOCs from food samples. This study shows that the low‐resolution Advion compact mass spectrometer possesses the requisite sensitivity and selectivity for food authenticity and safety testing when used in conjunction with the VOC sampling interface. The combination of this interface with a compact transportable mass spectrometer suggests this method has the potential for deployment in the field, enabling on‐site testing and reducing the need to send samples to specialised laboratories for analysis by high‐resolution methods, thus greatly improving the speed and throughput of analysis.

## Supporting information


**Figure S1**. APCI‐MS analysis of a 16.5 μg/ml indole standard in 50:50 MeOH/H_2_O + 0.1% Formic acid
**FIGURE S2.** Calibration curve of indole standard that demonstrates the quantitative capabilities of the system
**Table S1.** Variables list used in the cheese PCA‐X model.
**Figure S1**. APCI‐MS analysis of a 16.5 μg/ml indole standard in 50:50 MeOH/H_2_O + 0.1% Formic acid
**Figure S2**. S:N ratio measurements from 3 replicate APCI‐MS analyses of a 1 μg/ml indole standard in 50:50 MeOH/H_2_O + 0.1% Formic acid
