# Supplementary material for: Direct analysis of volatile organic compounds in foods by headspace extraction atmospheric pressure chemical ionisation mass spectrometry
Source: Rapid Commun Mass Spectrom. 2017 Oct 11;31(22):1947–56. doi: 10.1002/rcm.7975 (PMC5656932; doi:10.1002/rcm.7975)
Supplement: Supplementary file 1 — Figure S1. APCI‐MS analysis of a 16.5 μg/ml indole standard in 50:50 MeOH/H2O + 0.1% Formic acid FIGURE S2. Calibration curve of indole standard that demonstrates the quantitative capabilities of the system Table S1. Variables list used in the cheese PCA‐X model. Figure S1. APCI‐MS analysis of a 16.5 μg/ml indole standard in 50:50 MeOH/H2O + 0.1% Formic acid Figure S2. S:N ratio measurements from 3 replicate APCI‐MS analyses of a 1 μg/ml indole standard in 50:50 MeOH/H2O + 0.1% Formic acid [file RCM-31-1947-s001.docx]

Direct Analysis of Volatile Organic Compounds in Foods by Headspace Extraction Atmospheric Pressure Chemical Ionisation Mass Spectrometry

P. Perez-Hurtado,^a,b^ E. Palmer,^a^ T . Owen,^a^ C. Aldcroft,^b^ M.H. Allen,^b^ J. Jones,^c^ C. S. Creaser,^a^ M.R. Lindley,^d^ M.A. Turner,^a^ J.C Reynolds^a^†

^a^ Centre for Analytical Science, Department of Chemistry, Loughborough University, Ashby Road, Loughborough, Leicestershire LE11 3TU, UK

^b^ Advion Ltd. Kao Hockham Building, Edinburgh Way, Harlow, Essex CM20 2NQ UK

^c^ Advion Inc. 10 Brown Road Suite 101, Ithaca, NY 14850 USA

^d^ School of Sports, Exercise and Health Sciences, Loughborough University, Ashby Road, Loughborough, Leicestershire, LE11 3TU UK

†Corresponding author contact information:

Dr James Reynolds: [j.c.reynolds@lboro.ac.uk](mailto:j.c.reynolds@lboro.ac.uk); Tel - +44 (0)1509 222540

**Electronic Supplementary Information**

*m/z*

Counts

181.2

91.1

118.2

**Figure S1**. APCI-MS analysis of a 16.5 µg/ml indole standard in 50:50 MeOH/H_2_O + 0.1% Formic acid

**
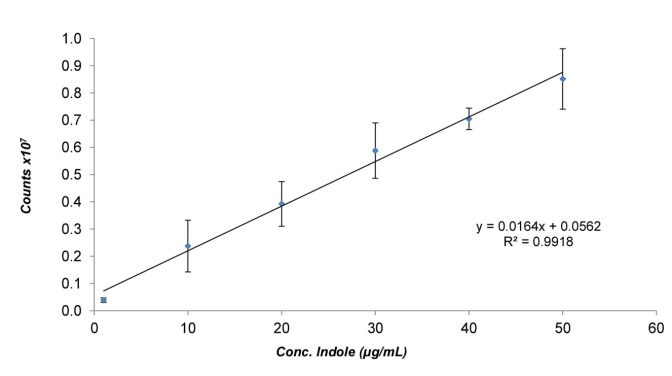
**

**FIGURE S2.** Calibration curve of indole standard that demonstrates the quantitative capabilities of the system

**Figure S2**. S:N ratio measurements from 3 replicate APCI-MS analyses of a 1 µg/ml indole standard in 50:50 MeOH/H_2_O + 0.1% Formic acid

**Table S1.** Variables list used in the cheese PCA-X model.

| Ion | Transformation | Equation |
| --- | --- | --- |
| 43 | Log | log_10_(x + 771175) |
| 43.05 | Log | log_10_(x + 272245) |
| 43.1 | Log | log_10_(x + 55242) |
| 43.15 | Log | log_10_(x + 607465) |
| 43.2 | Log | log_10_(x + 778982) |
| 43.25 | Log | log_10_(x + 243798) |
| 43.3 | Log | log_10_(x + 49389) |
| 43.35 | Log | log_10_(x + 20409.5) |
| 43.4 | Log | log_10_(x + 6493.5) |
| 43.45 | Log | log_10_(x + 19808) |
| 43.5 | Log | log_10_(x + 2751) |
| 43.55 | Log | log_10_(x + 3301.5) |
| 43.6 | Log | log_10_(x + 2476) |
| 43.65 | Log | log_10_(x + 275.5) |
| 43.7 | Log | log_10_(x + 1650.5) |
| 43.75 | Log | log_10_(x + 20221.5) |
| 43.8 | Log | log_10_(x + 11073.5) |
| 43.85 | Log | log_10_(x + 10454) |
| 43.9 | Log | log_10_(x + 56875) |
| 43.95 | Log | log_10_(x + 45859) |
| 44 | Log | log_10_(x + 26731) |
| 44.05 | Log | log_10_(x + 7669) |
| 44.1 | Log | log_10_(x + 6603) |
| 44.15 | Log | log_10_(x + 58576.5) |
| 44.2 | Log | log_10_(x + 49528) |
| 44.25 | Log | log_10_(x + 14373.5) |
| 44.3 | Log | log_10_(x + 2476) |
| 44.35 | Log | log_10_(x + 11554) |
| 44.4 | Log | log_10_(x + 8253.5) |
| 44.45 | Log | log_10_(x + 45952.5) |
| 44.5 | Log | log_10_(x + 101982) |
| 44.55 | Log | log_10_(x + 48116.5) |
| 44.6 | Log | log_10_(x + 74822) |
| 44.65 | Log | log_10_(x + 134034) |
| 44.7 | Log | log_10_(x + 176138) |
| 44.75 | Log | log_10_(x + 129243) |
| 44.8 | Log | log_10_(x + 2.16706e+006) |
| 44.85 | Log | log_10_(x + 1.95373e+006) |
| 44.9 | Log | log_10_(x + 1.00865e+006) |
| 44.95 | Log | log_10_(x + 4.08013e+006) |
| 45 | Log | log_10_(x + 5.03302e+006) |
| 45.05 | Log | log_10_(x + 2.50635e+006) |
| 45.1 | Log | log_10_(x + 1.5397e+006) |
| 45.15 | Log | log_10_(x + 1.23233e+006) |
| 45.2 | Log | log_10_(x + 3.06514e+006) |
| 45.25 | Log | log_10_(x + 2.88054e+006) |
| 45.3 | Log | log_10_(x + 1.24786e+006) |
| 45.35 | Log | log_10_(x + 618969) |
| 45.4 | Log | log_10_(x + 54334) |
| 45.45 | Log | log_10_(x + 92362) |
| 45.5 | Log | log_10_(x + 283941) |
| 45.55 | Log | log_10_(x + 131391) |
| 45.6 | Log | log_10_(x + 1.44531e+006) |
| 45.65 | Log | log_10_(x + 98796.5) |
| 45.7 | Log | log_10_(x + 2.11646e+006) |
| 45.75 | Log | log_10_(x + 2.8202e+006) |
| 45.8 | Log | log_10_(x + 4.25797e+006) |
| 45.85 | Log | log_10_(x + 241817) |
| 45.9 | Log | log_10_(x + 9.03835e+006) |
| 45.95 | Log | log_10_(x + 1.58405e+006) |
| 46 | Log | log_10_(x + 931583) |
| 58 | Log | log_10_(x + 17318.5) |
| 58.05 | Log | log_10_(x + 34904.5) |
| 58.1 | Log | log_10_(x + 38224) |
| 58.15 | Log | log_10_(x + 140202) |
| 58.2 | Log | log_10_(x + 139684) |
| 58.25 | Log | log_10_(x + 101028) |
| 58.3 | Log | log_10_(x + 14856) |
| 58.35 | Log | log_10_(x + 8484.5) |
| 58.4 | Log | log_10_(x + 18321.5) |
| 58.45 | Log | log_10_(x + 71145) |
| 58.5 | Log | log_10_(x + 13206) |
| 58.55 | Log | log_10_(x + 16094.5) |
| 58.6 | Log | log_10_(x + 708050) |
| 58.65 | Log | log_10_(x + 140482) |
| 58.7 | Log | log_10_(x + 327136) |
| 58.75 | Log | log_10_(x + 792112) |
| 58.8 | Log | log_10_(x + 899469) |
| 58.85 | Log | log_10_(x + 1.52454e+006) |
| 58.9 | Log | log_10_(x + 364637) |
| 58.95 | Log | log_10_(x + 3.45467e+006) |
| 59 | Log | log_10_(x + 1.68023e+006) |
| 59.05 | Log | log_10_(x + 228962) |
| 59.1 | Log | log_10_(x + 1.3945e+006) |
| 59.15 | Log | log_10_(x + 245349) |
| 59.2 | Log | log_10_(x + 143888) |
| 59.25 | Log | log_10_(x + 628024) |
| 59.3 | Log | log_10_(x + 2.04364e+006) |
| 59.35 | Log | log_10_(x + 538773) |
| 59.4 | Log | log_10_(x + 73681) |
| 59.45 | Log | log_10_(x + 52815) |
| 59.5 | Log | log_10_(x + 405661) |
| 59.55 | Log | log_10_(x + 1.44104e+006) |
| 59.9 | Log | log_10_(x + 262148) |
| 59.95 | Log | log_10_(x + 1.15951e+007) |
| 60 | Log | log_10_(x + 1.44617e+006) |
| 86 | Log | log_10_(x + 9904.5) |
| 86.05 | Log | log_10_(x + 1650.5) |
| 86.1 | Log | log_10_(x + 56352) |
| 86.15 | Log | log_10_(x + 1105.5) |
| 86.2 | Log | log_10_(x + 19326.5) |
| 86.3 | Log | log_10_(x + 3301.5) |
| 86.35 | Log | log_10_(x + 18641) |
| 86.4 | Log | log_10_(x + 19808.5) |
| 86.5 | Log | log_10_(x + 0.5) |
| 87.35 | Log | log_10_(x) |
| 87.5 | Log | log_10_(x + 745452) |
| 87.55 | Log | log_10_(x + 555280) |
| 88.55 | Log | log_10_(x + 4952) |
| 88.6 | Log | log_10_(x + 16569) |
| 88.65 | Log | log_10_(x + 71696.5) |
| 88.7 | Log | log_10_(x + 137226) |
| 88.75 | Log | log_10_(x + 124298) |
| 88.8 | Log | log_10_(x + 160897) |
| 88.85 | Log | log_10_(x + 54873) |
| 88.9 | Log | log_10_(x + 86799) |
| 88.95 | Log | log_10_(x + 64353.5) |
| 89 | Log | log_10_(x + 109536) |
| 89.05 | Log | log_10_(x + 98545) |
| 89.1 | Log | log_10_(x + 283018) |
| 89.15 | Log | log_10_(x + 39917.5) |
| 89.2 | Log | log_10_(x + 101901) |
| 89.25 | Log | log_10_(x + 135495) |
| 89.3 | Log | log_10_(x + 267317) |
| 89.35 | Log | log_10_(x + 72408.5) |
| 89.4 | Log | log_10_(x + 24280) |
| 89.45 | Log | log_10_(x + 3853.5) |
| 89.5 | Log | log_10_(x + 17312.5) |
| 89.55 | Log | log_10_(x + 23455) |
| 89.6 | Log | log_10_(x + 58483.5) |
| 89.65 | Log | log_10_(x + 59662) |
| 89.7 | Log | log_10_(x + 23798) |
| 89.75 | Log | log_10_(x + 1525) |
| 89.8 | Log | log_10_(x + 9354) |
| 89.85 | Log | log_10_(x + 4952) |
| 89.9 | Log | log_10_(x + 29986.5) |
| 89.95 | Log | log_10_(x + 11555) |
| 90 | Log | log_10_(x + 72861.5) |
| 100 | Log | log_10_(x + 99946.5) |
| 100.05 | Log | log_10_(x + 6602.5) |
| 100.1 | Log | log_10_(x + 17608) |
| 100.15 | Log | log_10_(x + 44733) |
| 100.2 | Log | log_10_(x + 32188) |
| 100.25 | Log | log_10_(x + 13564.5) |
| 100.3 | Log | log_10_(x + 6602) |
| 100.35 | Log | log_10_(x + 2888) |
| 100.4 | Log | log_10_(x + 72533) |
| 100.45 | Log | log_10_(x + 66903) |
| 100.5 | Log | log_10_(x + 6602.5) |
| 100.55 | Log | log_10_(x + 4539) |
| 100.6 | Log | log_10_(x + 1650.5) |
| 100.65 | Log | log_10_(x + 1375.5) |
| 100.7 | Log | log_10_(x + 110514) |
| 100.75 | Log | log_10_(x + 3301.5) |
| 100.8 | Log | log_10_(x + 353897) |
| 100.85 | Log | log_10_(x + 67953) |
| 100.9 | Log | log_10_(x + 149345) |
| 100.95 | Log | log_10_(x + 688172) |
| 101 | Log | log_10_(x + 1.01313e+006) |
| 101.05 | Log | log_10_(x + 200126) |
| 101.1 | Log | log_10_(x + 1.02425e+006) |
| 101.15 | Log | log_10_(x + 478695) |
| 101.2 | Log | log_10_(x) |
| 101.25 | Log | log_10_(x + 339214) |
| 101.3 | Log | log_10_(x + 67634) |
| 101.35 | Log | log_10_(x + 67835.5) |
| 101.4 | Log | log_10_(x + 138038) |
| 101.45 | Log | log_10_(x + 8728) |
| 101.5 | Log | log_10_(x + 7977.5) |
| 101.55 | Log | log_10_(x + 38324.5) |
| 101.6 | Log | log_10_(x + 53647) |
| 101.65 | Log | log_10_(x + 1651) |
| 101.7 | Log | log_10_(x + 8254) |
| 101.75 | Log | log_10_(x + 1650.5) |
| 101.8 | Log | log_10_(x + 2476) |
| 101.85 | Log | log_10_(x + 28371.5) |
| 101.9 | Log | log_10_(x + 6603) |
| 101.95 | Log | log_10_(x + 79658.5) |
| 102 | Log | log_10_(x + 14856.5) |
| 102.05 | Log | log_10_(x + 55088.5) |
| 102.1 | Log | log_10_(x + 23109.5) |
| 102.15 | Log | log_10_(x + 26061) |
| 102.2 | Log | log_10_(x + 11555) |
| 102.25 | Log | log_10_(x + 7978.5) |
| 102.3 | Log | log_10_(x + 19808) |
| 102.35 | Log | log_10_(x + 3576.5) |
| 102.4 | Log | log_10_(x + 26680) |
| 102.45 | Log | log_10_(x + 3301.5) |
| 102.5 | Log | log_10_(x + 18220) |
| 102.55 | Log | log_10_(x + 1375.5) |
| 102.6 | Log | log_10_(x + 1650.5) |
| 102.65 | Log | log_10_(x + 43857.5) |
| 102.7 | Log | log_10_(x + 2477) |
| 102.75 | Log | log_10_(x + 18205.5) |
| 102.8 | Log | log_10_(x + 1650) |
| 102.85 | Log | log_10_(x + 26525.5) |
| 102.9 | Log | log_10_(x + 18707.5) |
| 102.95 | Log | log_10_(x + 9905.5) |
| 103 | Log | log_10_(x + 32090) |
| 114 | Log | log_10_(x + 390.5) |
| 114.05 | Log | log_10_(x + 7428) |
| 114.1 | Log | log_10_(x + 11362.5) |
| 114.15 | Log | log_10_(x + 49559.5) |
| 114.25 | Log | log_10_(x + 3301) |
| 114.8 | Log | log_10_(x) |
| 114.85 | Log | log_10_(x) |
| 114.9 | Log | log_10_(x) |
| 114.95 | Log | log_10_(x) |
| 115 | Log | log_10_(x) |
| 115.05 | Log | log_10_(x) |
| 115.1 | Log | log_10_(x) |
| 115.15 | Log | log_10_(x) |
| 115.2 | Log | log_10_(x) |
| 115.25 | Log | log_10_(x) |
| 115.3 | Log | log_10_(x) |
| 115.4 | Log | log_10_(x) |
| 115.45 | Log | log_10_(x) |
| 115.5 | Log | log_10_(x) |
| 115.55 | Log | log_10_(x + 47159) |
| 115.6 | Log | log_10_(x + 161581) |
| 115.65 | Log | log_10_(x + 341300) |
| 115.7 | Log | log_10_(x + 65898) |
| 115.75 | Log | log_10_(x + 207548) |
| 115.9 | Log | log_10_(x + 171294) |
| 115.95 | Log | log_10_(x + 146303) |
| 116.1 | Log | log_10_(x) |
| 116.15 | Log | log_10_(x + 428753) |
| 116.2 | Log | log_10_(x + 173545) |
| 116.25 | Log | log_10_(x + 306903) |
| 116.3 | Log | log_10_(x + 120173) |
| 116.35 | Log | log_10_(x) |
| 116.45 | Log | log_10_(x + 53139) |
| 116.5 | Log | log_10_(x + 275564) |
| 116.6 | Log | log_10_(x + 26411) |
| 116.8 | Log | log_10_(x + 42332) |
| 116.85 | Log | log_10_(x + 79930.5) |
| 116.9 | Log | log_10_(x + 11602.5) |
| 116.95 | Log | log_10_(x + 75202) |
| 117 | Log | log_10_(x + 86537.5) |
| 141 | Log | log_10_(x + 28307) |
| 141.65 | Log | log_10_(x + 57668.5) |
| 141.95 | Log | log_10_(x + 49138) |
| 142 | Log | log_10_(x + 34251) |
| 142.05 | Log | log_10_(x + 19694.5) |
| 142.1 | Log | log_10_(x + 72079.5) |
| 142.15 | Log | log_10_(x + 13586.5) |
| 142.75 | Log | log_10_(x + 738590) |
| 142.8 | Log | log_10_(x) |
| 142.85 | Log | log_10_(x) |
| 142.9 | Log | log_10_(x) |
| 142.95 | Log | log_10_(x) |
| 143 | Log | log_10_(x) |
| 143.05 | Log | log_10_(x) |
| 143.1 | Log | log_10_(x) |
| 143.15 | Log | log_10_(x) |
| 143.2 | Log | log_10_(x) |
| 143.25 | Log | log_10_(x) |
| 143.3 | Log | log_10_(x) |
| 143.35 | Log | log_10_(x) |
| 143.4 | Log | log_10_(x) |
| 143.45 | Log | log_10_(x) |
| 143.5 | Log | log_10_(x) |
| 143.55 | Log | log_10_(x) |
| 143.6 | Log | log_10_(x) |
| 143.65 | Log | log_10_(x) |
| 143.7 | Log | log_10_(x + 352669) |
| 143.75 | Log | log_10_(x) |
| 143.8 | Log | log_10_(x + 133046) |
| 143.85 | Log | log_10_(x) |
| 143.9 | Log | log_10_(x) |
| 144 | Log | log_10_(x) |
| 144.05 | Log | log_10_(x) |
| 144.1 | Log | log_10_(x) |
| 144.15 | Log | log_10_(x) |
| 144.2 | Log | log_10_(x + 2.31886e+006) |
| 144.25 | Log | log_10_(x + 2.9744e+006) |
| 144.3 | Log | log_10_(x + 2.85988e+006) |
| 144.35 | Log | log_10_(x) |
| 144.4 | Log | log_10_(x + 301960) |
| 144.45 | Log | log_10_(x + 315156) |
| 144.5 | Log | log_10_(x + 147340) |
| 144.55 | Log | log_10_(x + 189186) |
| 145.1 | Log | log_10_(x + 896936) |
| 145.2 | Log | log_10_(x + 76465.5) |
| 145.75 | Log | log_10_(x + 3487) |
| 145.8 | Log | log_10_(x + 74969.5) |
| 145.85 | Log | log_10_(x + 13563.5) |
| 145.9 | Log | log_10_(x + 22284) |
| 145.95 | Log | log_10_(x + 123564) |
| 146 | Log | log_10_(x + 66309) |
| 158.05 | Log | log_10_(x + 12182) |
| 158.1 | Log | log_10_(x + 339657) |
| 158.15 | Log | log_10_(x + 211952) |
| 158.2 | Log | log_10_(x + 247503) |
| 158.25 | Log | log_10_(x + 231821) |
| 158.3 | Log | log_10_(x + 160523) |
| 158.35 | Log | log_10_(x + 577832) |
| 169.75 | Log | log_10_(x + 122974) |
| 170.6 | Log | log_10_(x + 1.70882e+006) |
| 170.65 | Log | log_10_(x) |
| 170.7 | Log | log_10_(x) |
| 170.75 | Log | log_10_(x) |
| 170.8 | Log | log_10_(x) |
| 170.85 | Log | log_10_(x) |
| 170.9 | Log | log_10_(x) |
| 170.95 | Log | log_10_(x) |
| 171 | Log | log_10_(x) |
| 171.05 | Log | log_10_(x) |
| 171.1 | Log | log_10_(x) |
| 171.15 | Log | log_10_(x) |
| 171.2 | Log | log_10_(x) |
| 171.25 | Log | log_10_(x) |
| 171.3 | Log | log_10_(x) |
| 171.35 | Log | log_10_(x) |
| 171.4 | Log | log_10_(x) |
| 171.45 | Log | log_10_(x) |
| 171.5 | Log | log_10_(x) |
| 171.55 | Log | log_10_(x) |
| 171.6 | Log | log_10_(x) |
| 171.65 | Log | log_10_(x) |
| 171.7 | Log | log_10_(x) |
| 171.75 | Log | log_10_(x) |
| 171.8 | Log | log_10_(x + 745042) |
| 171.85 | Log | log_10_(x + 270774) |
| 171.9 | Log | log_10_(x + 3.4104e+006) |
| 171.95 | Log | log_10_(x + 3.06083e+006) |
| 172 | Log | log_10_(x + 1.51368e+006) |
| 172.05 | Log | log_10_(x + 2.67713e+006) |
| 172.15 | Log | log_10_(x + 2.58311e+006) |
| 172.2 | Log | log_10_(x + 3.93012e+006) |
| 172.25 | Log | log_10_(x + 2.4587e+006) |
| 172.3 | Log | log_10_(x) |
| 172.35 | Log | log_10_(x) |
| 172.4 | Log | log_10_(x) |
| 172.45 | Log | log_10_(x) |
| 172.55 | Log | log_10_(x + 751213) |
| 200.7 | Log | log_10_(x + 104222) |
| 200.75 | Log | log_10_(x + 45052.5) |
| 200.8 | Log | log_10_(x + 6232) |
